# Supplementary material for: Short sleep duration is a significant risk factor of obesity: A multicenter observational study of healthy adults in Japan
Source: PLoS One. 2025 Mar 5;20(3):e0319085. doi: 10.1371/journal.pone.0319085 (PMC11882098; doi:10.1371/journal.pone.0319085)
Supplement: Attachment — Submitted filename: Response to Reviewers 20250103_Yamamichi.docx [file pone.0319085.s002.docx]

Dear Editor of PLoS ONE

We are grateful to resubmit our manuscript (PONE-D-24-37623) entitled “Short sleep duration is a significant risk factor of obesity: A multicenter observational study of healthy adults in Japan”. According to the associate editor and reviewers’ instructions, we have revised our manuscript as follows.

**Response to the Journal Requirements or comments of the associate editor**

Journal Requirements:

When submitting your revision, we need you to address these additional requirements.

1. Please ensure that your manuscript meets PLOS ONE's style requirements, including those for file naming. The PLOS ONE style templates can be found at

https://journals.plos.org/plosone/s/file?id=wjVg/PLOSOne_formatting_sample_main_body.pdf and

https://journals.plos.org/plosone/s/file?id=ba62/PLOSOne_formatting_sample_title_authors_affiliations.pdf

According to the requirement, we have revised our manuscript on the basis of PLOS ONE’s style. In the revised manuscript, we have changed the order of section as follows; Abstract, Introduction, Methods, Results, Discussion, and Conclusions, Acknowledgements, and References.

2. We note that the grant information you provided in the ‘Funding Information’ and ‘Financial Disclosure’ sections do not match.

When you resubmit, please ensure that you provide the correct grant numbers for the awards you received for your study in the ‘Funding Information’ section.

According to the requirement, we have checked and corrected the grant we used for this study.

3. Thank you for stating the following financial disclosure:

“This study was supported by Grant-in-Aid for Early-Career Scientists (no. 20K19203), Grant-in-Aid for Scientific Research (C) (no. 22K11255), and Grant-in-Aid for Challenging Exploratory Research (no. 22K19665).”

Please state what role the funders took in the study. If the funders had no role, please state: "The funders had no role in study design, data collection and analysis, decision to publish, or preparation of the manuscript." If this statement is not correct you must amend it as needed.

Please include this amended Role of Funder statement in your cover letter; we will change the online submission form on your behalf.

In the covering letter for revised manuscript, we have added the comment as follows.

Concerning the financial disclosure, our study was supported by Grant-in-Aid for Early-Career Scientists of Japan Society for the Promotion of Science (no. 20K19203), Grant-in-Aid for Scientific Research (C) of Japan Society for the Promotion of Science (no. 22K11255), and Grant-in-Aid for Challenging Exploratory Research of Japan Society for the Promotion of Science (no. 22K19665). The funders had no role in study design, data collection and analysis, decision to publish, or preparation of the manuscript.

4. In this instance it seems there may be acceptable restrictions in place that prevent the public sharing of your minimal data. However, in line with our goal of ensuring long-term data availability to all interested researchers, PLOS’ Data Policy states that authors cannot be the sole named individuals responsible for ensuring data access (http://journals.plos.org/plosone/s/data-availability#loc-acceptable-data-sharing-methods).

Data requests to a non-author institutional point of contact, such as a data access or ethics committee, helps guarantee long term stability and availability of data. Providing interested researchers with a durable point of contact ensures data will be accessible even if an author changes email addresses, institutions, or becomes unavailable to answer requests.

Before we proceed with your manuscript, please also provide non-author contact information (phone/email/hyperlink) for a data access committee, ethics committee, or other institutional body to which data requests may be sent. If no institutional body is available to respond to requests for your minimal data, please consider if there any institutional representatives who did not collaborate in the study, and are not listed as authors on the manuscript, who would be able to hold the data and respond to external requests for data access? If so, please provide their contact information (i.e., email address). Please also provide details on how you will ensure persistent or long-term data storage and availability.

The data underlying the results presented in the study are available from the corresponding author of the manuscript ([nobutakeyamamichi@gmail.com](mailto:nobutakeyamamichi@gmail.com), a private email of corresponding author). In addition, the data are also available from the Center for Epidemiology and Preventive Medicine, The University of Tokyo Hospital via e-mail (dock@h.u-tokyo.ac.jp, an officially used email of our medical institute). Neither the ethics committee nor other third party organizations do not share with our data.

5. Your ethics statement should only appear in the Methods section of your manuscript. If your ethics statement is written in any section besides the Methods, please move it to the Methods section and delete it from any other section. Please ensure that your ethics statement is included in your manuscript, as the ethics statement entered into the online submission form will not be published alongside your manuscript.

We moved the ethics statemen in the end of the Methods.

6. Please include captions for your Supporting Information files at the end of your manuscript, and update any in-text citations to match accordingly. Please see our Supporting Information guidelines for more information: http://journals.plos.org/plosone/s/supporting-information.

We have uploaded the two ethical approvement documents concerning this study (Ethical approvement of Kameda Medical Center and University of Tokyo Hospital) as supporting information. Therefore, we added the two captions for Supporting Information files.

**Response to the Reviewers’ Comments**

**Reviewer #1:**

1.The study used healthy adults who came for a checkup. How did authors define a healthy population? How was the unhealthy population excluded? The exclusion criteria were not mentioned in the method section or Figure 1.

In this study, we did not perfectly exclude the people with some latent diseases or disorders. Therefore, we defined them as “generally healthy people”. In the health checkup, few of participants (“generally healthy people”) are finally diagnosed as illness or some disease. In other words, the percentage of people with some latent diseases or disorders was very low in our study population. In the “Study participants” of the Method section (page 5, line 80-85), we added the detailed explanation of Japanese health checkup.

2.The study lacks data on ethnic groups within the population. The authors mention that the findings are relevant to an Asian demographic. Yet, the population in this study is likely dominated by East Asians. Please provide basic information on the ratios of other ethnic groups like South Asian, Caucasian, and Black.

We are grateful to this important comment. Unfortunately, however, we did not investigate the nationality of the participants. It was because almost all of them have Japanese nationality in the health checkup in Japan. As a result, our results certainly reflect the data of sleep in Japan and therefore in East Asia. We added it as one of the limitations in the Discussion section (page 24, line 430 - 433).

3.Follow-up study: How long did the authors monitor the population? This needs clarification. Was the follow-up period sufficient to identify HR, particularly for glucose intolerance? The event rate for glucose intolerance is lower compared to the other two.

Thank you for this useful comment. We performed longitudinal analyses of the 31,004 participants for the development of obesity, 39,048 participants for the development of impaired glucose tolerance, and 33,537 participants for the development of hypertriglyceridemia. These have already described in the manuscript. The total observation period was 11 years (from 2010 to 2020). We newly added this sentence in the revised manuscript (page 16, line 285 - 286).

4.Quantitative data in Table 2 are not defined as mean ± SD, which should be detailed in the methods section.

Thank you for the comment. According to the comment, in the Methods section, we added the sentence: The above 1)-10) factors were quantitative data and shown as mean ± standard deviation (SD) in the table (page 7 line 119-120). In addition, we changed the caption of Table 2 from “the mean” to “the mean (± SD)”.

**Reviewer #2:**

1. The manuscript is technically sound, and the data do support the conclusions.

2. The statistical analysis has been performed appropriately and rigorously.

3. The authors have made all data underlying the findings in their manuscript fully available.

4. The manuscript is presented in an intelligible fashion and written in standard English.

We were gratefully encouraged by these comments. There are no corrections based on these comments.

**Reviewer #3:**

1.The methodology is not clear. I suggest the authors to provide more detail about the medical check-up, the facility care of the study participants had been evaluated, the laboratory measurements (in a central or in various centers), the inclusion and exclusion criteria in more detail

Thank you for this important comment. About the health checkup, we added the detailed explanation of Japanese health checkup in the “Study participants” of the Method section (page 5, line 80 - 85).

About the data collection, we added the sentence as follows. All the data concerning laboratory measurements or life-style related factors were separately derived from the participated medical institutes and finally merged (page 5, line 88 - 90).

2.Does Table 2 identify baseline characteristics of the study participants?

Yes, it does. Table 2 shows the baseline characteristics of 62,056 study participants. It also shows the association between background factors and sleep based on the univariate analysis.

3.How many of the study participants were shift-workers?

Unfortunately, we have no data about the work of participants. We added it as one of the limitations of our study (page 23, line 427).

4.How many of the study participants had thyroid dysfunctions?

5.How many of the study participants had been diagnosed with chronic diseases (hypertension, diabetes, dyslipidemia)?

6.How many of the study participants had been on sleep modifier medications, including antidepresants, antihistaminics, etc?

8.Did the authors have data on menopause status of women participants? This may have impact on sleep duration

9. How many of the participants had been on hypolipidemic, antihypertensive drugs when evaluated longitudinally.

The study participants in our study were “generally healthy people” in Japan, who underwent comprehensive health checkup in the medical institutes. In Japan, those with some disease usually receive medical treatment at hospitals or clinics, cost of which is covered by health insurance. On the contrary, generally healthy people (who do not usually take medications) annually go to the medical institute for body health checkup. In this study, we analyzed the latter generally healthy people (without apparent diseases) who underwent annual comprehensive health checkup in the medical institutions. The aim of such body health checkup is to find the latent disease or hidden health problems among the generally healthy people. We added the description in the “Study participants” of the Method section (page 5, line 80 - 85).

Therefore, almost all of the study participants did not have thyroid dysfunctions, had not been diagnosed with chronic diseases, did not have been on sleep modifier medications, and were not suffering from menopausal disorder. Nevertheless, it is true that a few unhealthy people taking some medications inappropriately undergo such health checkup. Due to the study design, we could not omit such inappropriate cases from our study participants, which was already described in the limitation of our study. However, as described above, the percentage of such people with some diseases was low in the health checkup in Japan.

7. Did the authors have data related to family history of diabetes, dyslipidemia, etc?

We did not have any data related to family history, which were not described in the study protocol.

10. I suggest the authors to include triglyceride to HDL-C ratio as a surrogate marker of insulin resistance and may be considered a reliable marker for such a study.

We are very grateful to this important comment. However, we cannot add the “triglyceride to HDL-C ratio” as one of the observation items, because it is not included in the study protocol which was approved by the ethic committee. We will try to use it in the next study which we are now planning based on the results of this study.

11. In Table 2, I suggest the authors to provide the percentages of age in parenthesis.

We are grateful to this important comment. According to this indication, we have added the percentage for age in Table 2. Aligning with it, we have also added the percentage for BMI in Table 2. All the corrections in Table 2 were highlighted in yellow in the revised manuscript.

12. In Discussion, it should be emphasized that most of the study population had normal BMI, and the obese patients constituted the minority. Such a population may not reflect the importance of the association between sleep duration and the investigated parameters.

Thank you for the comment. However, as shown in Table 2, the percentage of abnormally high BMI in our study population was not so small. In the 34,239 male participants, 10,778 (474 + 2,336 + 4,479 + 2,609 + 880) men were diagnosed as obesity (31.4%). In the 27,817 female participants, 4,076 (223 + 1,137 + 1,632 + 823 + 261) women were diagnosed as obesity (14.7%). We think it was not true that most of our study population had normal BMI.

**Reviewer #4:**

Please define what is habitual drinking and also habitual exercise?

Thank you for the comment. However, in the "Observation items as explanatory or objective variables" in the Methods section, we have already described the definition of habitual drinking and habitual exercise as follows.

12) habitual drinking (“drinking three or more days per week” or not)

13) habitual exercise (“at least 30 min at once and at least three times a week” or not)

In the discussion, can authors elaborate on the physiological mechanism of sleeping as mentioned.

Thank you very much for this fruitful comment. Accordingly, we have added the description in the Discussion section (page @@, line @@ - page @@, line @@).
